# Evaluation of the quality of life among elderly female athletes

**DOI:** 10.1590/S1516-31802006000500014

**Published:** 2006-09-07

**Authors:** Guilherme Turolla Sguizzatto, Luiz Eugênio Garcez-Leme, Luciana Casimiro

**Keywords:** Quality of life, Motor activity, Sports, Depression, Aging, Qualidade de vida, Atividade física, Esportes, Depressão, Envelhecimento

## Abstract

**OBJECTIVE:**

To analyze quality of life (QOL) in elderly athletes.

**DESIGN:**

Transversal, controlled study.

**METHODS:**

Female athletes over 60 years who run 15 km, and a control group consisting of 15 healthy, sedentary, women over 60 were studied. Questionnaires on QOL and Depression were applied.

**RESULTS:**

Athletes show better functional capacity (98.8 versus 73.3), less pain (90.6 versus 64.9), better general state of health (86.8 versus 66.8) and better vitality (86.2 versus 67.3). Differences were observed in emotional characteristics (89.6 versus 60.0) and mental health (84.3 versus 68.3), with fewer depressive answers (1.9 versus 3.8).

**CONCLUSION:**

Regular physical activity was related to better quality of life.

## RESUMO

**OBJETIVO:**

Analisar qualidade em vida de atletas idosas.

**TIPO DE ESTUDO:**

Estudo transversal controlado.

**MÉTODOS:**

Atletas maiores de 60 anos, que correm 15 km, e grupo controle com 15 mulheres saudáveis, sedentárias foram estudados. Aplicados questionários de qualidade de vida e depressão.

**RESULTADOS:**

A capacidade funcional das atletas foi maior (98,8 *versus* 73,3). As atletas referiram menos dor (90,6 *versus* 64,9), melhor condição geral de saúde (86,8 *versus* 66,8), melhor vitalidade (86,2 *versus* 67,3). Houve diferença no aspecto emocional (89,6 *versus* 60,0) e saúde mental (84,3 *versus* 68,3), com menos respostas depressivas (1,9 *versus* 3,8).

**CONCLUSÃO:**

A atividade física regular foi relacionada com melhor qualidade de vida.

Regular physical activity is seen as one of the most effective procedures for promoting quality of life in any population.^1,2^ The relationship between physical activity and depression in the elderly population presents controversial aspects, with epidemiological studies suggesting a diverse relationship between the amount of activity and the depressive symptoms, in which these symptoms increase in individuals who report both low and high levels of physical activity.^3^

Women perform less energetic leisure activities than men and, on average, with shorter duration. In all age groups, women participate in sports or physical leisure activities less frequently.^4^

From this perspective, we analyzed the quality of life (Short Form-36, SF-36,^5^ and Geriatric Depression Scale-15, GDS-15^6^) of elderly female athletes in high performance competitions (the annual São Silvestre race, in the city of São Paulo), by comparing these individuals' quality of life with that of controls matched for sex and age. This was a preliminary cross-sectional study with controlled observations for evaluating and comparing the quality of life and the presence of depressive symptoms among women aged 60 years and over. The subjects were 31 female volunteers: 16 athletes and 15 healthy sedentary women with no complaints, pain symptoms or physical limitations.

To compare the sedentary and athletic groups, we used Student's t test for the variables presenting parametric distributions and the Mann-Whitney U-test for the non-parametric distributions. The 5% confidence level was adopted (α = 0.05).

The study group (athletes) and the control group (sedentary women) were shown to be homogeneous, with no significant differences in terms of age, physical characteristics and social characteristics.

There were significant differences in functional capacity, pain, general health condition, vitality, emotional characteristics, mental health and Geriatric Depression Scale scores (Table 1). From this preliminary study, we concluded that regular physical activity and high performance sports were related to better quality of life and fewer depressive symptoms among elderly women and that this could be a tool for promoting physical and mental health.

Furthermore, we concluded that more structured studies, such as specific cohorts, are justified in order to answer the remaining questions regarding high-performance sports and aging.

**Table 1:** Results from investigations on athletes and controls, and their significance

	Athletes		Controls	p
**Age**		66.2	67.5	0.22
**SF-36**	Functional capacity	98.8	73.3	2.7 × 10^−6^*
	Physical characteristics	97.8	88.3	0.59
	Pain	90.6	64.9	0.01*
	General state of health	86.8	66.8	0.0001*
	Vitality	86.2	67.3	0.0004*
	Social characteristics	90.6	75.0	0.06
	Emotional characteristics	89.6	60.0	0.02*
	Mental health	84.3	68.3	4.9 × 10^−5^*
**GDS-15**		1.9	3.8	0.03*

* = Statistically significant; SF-36 = Short Form-36; GDS = Geriatric Depression Scale.
